# Number of Heat Wave Deaths by Diagnosis, Sex, Age Groups, and Area, in Slovenia, 2015 vs. 2003

**DOI:** 10.3390/ijerph15010173

**Published:** 2018-01-22

**Authors:** Simona Perčič, Andreja Kukec, Tanja Cegnar, Ana Hojs

**Affiliations:** 1 Centre for Environmental Health, National Institute of Public Health Slovenia, Zaloška 29, 1000 Ljubljana, Slovenia; ana.hojs@nijz.si; 2 Department for Public Health, Medical Faculty, University of Ljubljana, Zaloška 4, 1000 Ljubljana, Slovenia; andreja.kukec@mf.uni-lj.si; 3 Slovenian Environment Agency, Vojkova cesta 1b, 1000 Ljubljana, Slovenia; tanja.cegnar@gov.si

**Keywords:** heat waves, excess deaths, vulnerabilities, cause of death, sex, age group, area

## Abstract

*Background*: Number of deaths increases during periods of elevated heat. *Objectives*: To examine whether differences in heat-related deaths between 2003 and 2015 occurred in Slovenia. *Materials and Methods*: We estimated relative risks for deaths for the observed diagnoses, sex, age, and area, as well as 95% confidence intervals and excess deaths associated with heat waves occurring in 2015 and 2003. For comparison between 2015 and 2003, we calculated relative risks ratio and 95% confidence intervals. *Results*: Statistically significant in 2015 were the following: age group 75+, all causes of deaths (RR = 1.10, 95% CI 1.00–1.22); all population, circulatory system diseases (RR = 1.14, 95% CI 1.01–1.30) and age group 75+, diseases of circulatory system (RR = 1.17, 95% CI 1.01–1.34). Statistically significant in 2003 were the following: female, age group 5–74, circulatory system diseases (RR = 1.69, 95% CI 1.08–2.62). *Discussion*: Comparison between 2015 and 2003, all, circulatory system diseases (RRR = 1.25, 95% CI 1.01–1.55); male, circulatory system diseases (RRR = 1.85, 95% CI 1.41–2.43); all, age group 75+ circulatory system diseases (RRR = 1.34, 95% CI 1.07–1.69); male, age group 75+, circulatory system diseases (RRR = 1.52, 95% CI 1.03–2.25) and female, age group 75+, circulatory system diseases (RRR = 1.43, 95% CI 1.08–1.89). *Conclusions*: Public health efforts are urgent and should address circulatory system causes and old age groups.

## 1. Introduction

Many published epidemiological studies have noted increased mortality due to different causes (diagnosis) in summer during extreme elevated weather temperature episodes, which are usually defined as heat waves [1,2,3,4,5]. Heat wave mortality has been studied intensely in the last two decades after well-known disasters—such as in the case of Chicago in July 1995 [6] and in France in August 2003 [7]. The summer of 2003 was considered the hottest in Europe since 1500 [8]. That same year, during summer, extreme heat waves occurred all over Europe resulting in around 22,000 to 70,000 excess deaths [8]; calculated specifically for this part of the world [9]. The first and foremost problem is that over the next century, heat waves will become more frequent, more intense, and will only last longer [10]. This climate change phenomenon over the next century is also shown in projections for Slovenia [11]. The second problem is that increased longevity is changing society’s demographics. The forecasts predict ageing of population, with a lengthening of life expectancy almost in every European country, particularly in urban areas. By 2050 21.1% of population in Europe is expected to be older than 60 [12].

Many previous studies have successfully identified vulnerable populations with limited adaptation resources to extreme temperatures during heat waves, which are especially of concern as they contribute to the burden of disease and premature deaths [13]. Age is a very well-known risk factor for increased mortality during heat waves [14] due to physiological changes that occur with ageing, worse access to medical care, chronic illness, certain medications, sedentary lifestyles, and the lack of availability of air-conditioning. These factors all affect body temperature regulation and consequently lead to dehydration [15]. Polypharmacy is one of the problems of old age, and additionally, several drugs can worsen the effects of heat waves and accelerate dehydration; including diuretics, serotonergic antidepressants, angiotensin-converting inhibitors, proton pump inhibitors, non-dopaminergic anti-Parkinsonians or antiepileptic drugs, and beta-blockers [16]. Other vulnerable populations include those with cardiovascular [17], respiratory [9,18] and renal diseases [9,18], diabetes [17], those with mental health problems [19,20], overweight and pre-existing diseases [21]. Many studies have identified other social risk factors: living alone, living on the top floor, lack of air-conditioning, socioeconomic situation, working outdoors, and living in heat islands in the cities, where temperatures do not drop significantly even during the evening and at night [21,22,23]. The vulnerable subgroup includes women, although some reports disagree; this is probably associated with cultural, religious habits and socio-economic status [2,24,25]. Changes in the susceptibility of a population to heat may occur at shorter time scales, for example, within seasons [26].

On the basis of recent research, the WHO and other international institutions already started with some recommendation how to adapt to climate change globally with changes in urban design, more energy efficient buildings, education and outreach to vulnerable population. What is important, interventions which deal with medical, social and environmental public health are already designed and include general populations as well as vulnerable populations [27,28]. However, to implement these recommendations, there is a growing need for researchers to identify local and regional heat-related health impacts and for them to assess regional and local vulnerabilities [27,29].

The aims of our study were first to identify which vulnerable populations for increased mortality during heat waves are especially relevant for Slovenia. The second was to compare two years; the hottest in the first decade in 2000, and the hottest since 2010. We were interested in seeing if the mortality increased with years, due to climate change, as the problems with this phenomenon is growing all over the world. Provided evidences have important implications for the future work in the field of climate change and public health in Slovenia.

## 2. Materials and Methods

### 2.1. Study Area

Republic of Slovenia is a nation state in Southern Central Europe. It had population of 1,996,773 in 2003, and of 2,062,874 in 2015 [30,31]. Slovenia’s meteorological network covers an area of over 20,000 square km (7827 sq. mi) [32]. There are four major climate systems: littoral Slovenia, central Slovenia, the more continental part in the northeastern Slovenia and alpine Slovenia (the mountainous region). The territory of Slovenia strongly influences the country’s weather. It is mainly hilly, with the exception of the Slovene littoral and the north-western area occupied by the Alps [33].

### 2.2. Number of Deaths Source

Records of all underlying causes of deaths by age group, sex, and municipality for May through September 2003 and 2015 were obtained from the National Institute of Public Health (NIPH) Mortality database, using the International Classification of Diseases, Tenth Revision (ICD 10).

### 2.3. Definition of Heat Wave, Heat Wave Deaths and Expected Deaths

To assess heat load, we used pseudo-equivalent temperatures calculated based on daily maximum temperatures and water vapour pressure in the early afternoon. An average value from a few monitoring stations was used; stations are representative for three different climatic regions in Slovenia (littoral Slovenia, central Slovenia, and the more continental part in the northeastern Slovenia) [33]. We are dealing with a relatively small sample. Several studies in the past has shown that a sample to be statistical representative must be big enough, in some studies carried out at the beginning by an expert group under the umbrella of the World Meteorological Organization it was recommended (see also PHEWE methodology and city selection criteria) that the sample should be at least 1 million. In Slovenia there is no such large cities, therefore we used mortality data from the whole county and in order to keep consistency we also had to aggregate the value of a measure describing meteorological condition [34]. Description of this aggregation is provided bellow.

Based on the previous studies and methodology adopted in compilation of environment indicator “Heat waves and daily number of deaths” in group of indicators describing “Human health and ecosystem resilience” the average value of three stations representative for the most populated climatic regions in Slovenia has been used. These stations are Bilje (Nova Gorica) on the west of Slovenia, Ljubljana (the capital and representative for the central region of Slovenia), and Murska Sobota (situated on the plain terrain in northeast Slovenia). The mountainous regions is scarcely populated and heat stress is extremely rare or completely absent in the mountains, consequently the mountainous region of Slovenia has not been taken into consideration in this study [35].

A heat wave was defined as a period of two or more consecutive days with a pseudo-equivalent daily temperature exceeding 56 °C. Pseudo-equivalent daily temperature was calculated as the sum of air temperature and 1.5 multiplied by the partial pressure of water vapour [36]. Temperature data were obtained from the Slovenian Environment Agency which is a body of the Ministry of the Environment and Spatial Planning.

Based on the previous studies and methodology adopted in compilation of environment indicator “Heat waves and daily number of deaths” in groups of indicators describing “Human health and ecosystem resilience” the above mentioned time lag was used [35].

We used the term pseudo-equivalent temperature as air temperature doesn’t take into account air humidity, which is an important factor when assessing heat load. In order to assess a combined effect of air temperature and humidity on thermal discomfort in hot and sultry conditions an index combining air temperature and humidity has been used in Slovenia already for several decades [36].

The heat wave periods in our study were all heat waves which occurred between the period of 1 May to 30 September in the years 2015 and 2003 in Slovenia. In 2015, this included seven heat wave periods, and in 2003, this included 11 heat wave periods defined by a previous meteorological analysis. In 2015, the heat wave period lasted for 39 days and in 2003 for 31 days. See Table 1 for details. We defined heat wave deaths and reference days’ deaths as any death by the ICD 10 occurring during heat wave and reference days’ period.

The reference day periods were defined as in previous study [37,38,39]. The reference day periods for 2015 were drawn from the same summer (from 20 May to 2 June, from 5 June to 1 July, from 9 to 13 July, from 26 July to 1 August, from 9 to 13 August, from 17 to 26 August, from 1 to 15 September, and from 19 to 23 September) as it was done in previous analyses [39]; to calculate all together: 78 days without heat waves. The same was done for 2003 (from 1 May to 5 June and from 1 September to 26 September); to calculate all together: 62 days without heat waves. We adopted the methodology from Joe and co-workers [39] to select the number for the reference days: days of heat waves multiplied by 2 to increase statistical precision. The number of reference days before and after the heat waves periods for both years was approximately the same.

As far as so called “lag” and “harvesting” effects are concerned, previous studies examined that one should consider these two effects when defining the heat wave period and the reference period. A time period lasting from 0 to 3 days [1,40,41] was proposed, or even longer in other similar studies [42,43]. Due to these facts we did not included the days immediately after the heat wave in the reference periods. For the heat waves we defined 0 day of “lag” or/and “harvesting”.

### 2.4. Definitions of Subgroups

To examine risks associated with causes (diagnosis) of deaths, we calculated RRs for dying because of all causes (ICD 10, A00–T98): circulatory system diseases (ICD 10, I00–I99), respiratory system diseases (ICD 10, J00–J99), endocrine diseases (ICD 10, E00–E90), digestive system diseases (ICD 10, K00–K93), genitourinary system diseases (ICD 10, N00–N99) and neoplasms (ICD 10, C00–D48).

For sex, we calculated RRs separately for males and females dying of all causes, circulatory system diseases and respiratory system diseases.

For age groups, we also decided to define three age subgroups: from 0 to 4 years, from 5 to 74 years, and 75+ years. As there are small numbers of deaths in one day in Slovenia, we excluded the age group from 0 to 4 years, since during the whole observational time there was only one death reported in this age group. For the other two age groups, we calculated RRs separately for dying of all causes, circulatory system diseases, and respiratory system diseases; separately for males and females.

Urban versus rural areas in Slovenia were difficult to define, since we have only two cities which exceed 100,000 inhabitants; namely the capital Ljubljana and the city of Maribor. In the research, we included these two municipalities as urban areas, and all other fifty-six municipalities as rural areas.

### 2.5. Statistical Analysis: Relative Risk, Excess Deaths, and Relative Risks Ratio

All the data was prepared and analysed using Microsoft Excel, version 2010 (Microsoft, Redmond, WA, USA). Like past studies [37,38,39], we adopted a simplified relative risk (RR) approach, comparing deaths occurring during heat wave periods and reference day periods [37,38,39]. We presumed that population during one observed year remains constant as was done in previous studies [37,38,39]. We calculated the RR to assess the effects of the heath waves by dividing the number of deaths during the heat wave periods (*A*_1_) by the number of deaths during the reference days (*A*_0_), divided by two, since we selected for the reference days the number of heat wave days multiplied by 2 [39], (Equation (1)):

(1) $$RR=\frac{A_{1}}{\frac{A_{0}}{2}}$$

We calculated 95% confidence intervals (95% CI) for the RR using a simple approach for person-time incidence ratios (Equation (2)) [39]:

(2) $$95\%\;\mathrm{CI}=e^{\ln RR\pm 1.9\sqrt[{6}]{\frac{1}{A_{1}}+\frac{1}{A_{0}}}}$$

Statistically significant increases of deaths during heat waves were in the subgroups where the lower limit of the 95% CI was above 1.00. To assess excess deaths, we adopted the already described method by Joe and co-workers [39] (Equation (3)):

(3) $$\mathrm{Excess}\;\mathrm{Deaths}=A_{1}-\frac{A_{0}}{2}$$

To compare RRs between subcategories in 2015 and 2003, a method previously described by Altman was adopted [44]. We calculated RRRs (the difference between two estimates) and 95% confidence intervals (95% CI), to see whether the statistically significant differences between two seasons even occurred.

## 3. Results

The recorded values of the average pseudo-equivalent temperatures affected Slovenia between 1 May and 30 September in the years 2003 and 2015. In 2015, the pseudo-equivalent temperature record reached its peak on 23 July with 69.7 °C, and on 18 August, in 2003 with 64.6 °C. In 2015, higher average of the average pseudo equivalent temperatures occurred in comparison with 2003 (Table 1).

### 3.1. Analysis of Heat Waves in 2015

Overall in 2015, there were 137 (7%) more deaths than expected in Slovenia during the heat wave periods, but this was not statistically significant. Of these excess deaths 98 (14%) occurred due to circulatory diseases (all circulatory system diseases RR = 1.14, 95% CI 1.01–1.30) and none due to respiratory diseases. Deaths that occurred due to circulatory system diseases, were the only ones which had a statistically significant role in our research. Examining deaths occurring due to other diagnosis reveal excess deaths in all the observed diagnosis subgroups, except in digestive system disease. However none of them were significant: endocrine disease: 7 (19%), digestive system diseases, genitourinary system diseases: 9 (36%) and neoplasms: 33 (5%) (Table 2).

In examining deaths by sex in 2015, we discovered there were overall 52 (6%) more deaths than expected during heat waves in the male population, and a slightly higher percentage inside the female population at 89 (10%) more deaths than expected during heat waves. This is insignificant for both the female and male population. In examining deaths occurring in males due to circulatory diseases, we discovered there were 42 (15%) more deaths and 56 (13%) more female deaths than expected during the heat waves. This is also an insignificant percentage for both sexes. Due to confidence intervals being too wide it is hard to conclude any noteworthy differences between the two statistical subgroups; males and females (Table 2). The two (4%) excessive respiratory disease induced deaths among males and none such death among the females were disregarded as well (Table 2).

An examination of overall deaths by age groups in 2015 revealed 22 (4%) more deaths than expected during heat waves in the 5–74 age group. In the age group 75+ the value was slightly higher, with 115 (10%) more deaths than expected (RR = 1.11, 95% CI 1.00–1.22), which holds significance for the 75+ age group. An examination of deaths occurring in the age group 5–74 due to circulatory diseases revealed six (4%) more deaths than expected during the heat waves. A much higher percentage can be found in the 75+ age group with the number of deaths exceeding the expected amount by 92 (17%) deaths (RR = 1.17, 95% CI 1.01–1.34). This is again, statistically significant for the 75+ age group. Analysis of data pertaining to deaths among patients with respiratory diseases showed no excess deaths in the 5–74 age group and only four more deaths than expected in the 75+ age group, which is an insignificant ratio for both subgroups. While examining deaths occurring in the 5–74 age group due to circulatory diseases for males 12 (14%) more deaths than expected occurred during the heat waves and 24 (11%) deaths exceeding the expected number of deaths was noted for males in the 75+ age group. This is insignificant for both of the aforementioned age groups. In examining deaths occurring in the 5–74 age group due to circulatory diseases in females, no more deaths than expected during the heat waves occurred, and in the 75+ age group the 62 (17%) more deaths than expected during the heat waves occurred. It is not significant neither for the 5–74 age group, nor for the 75+ age group. Confidence intervals are too wide to conclude anything about differences between subgroups; excess mortality in the 5–74 age group and the 75+ age group (Table 2). The data about excessive number of deaths because of respiratory system diseases during heatwaves gathered for the age group of 5–74 revealed no unexpected fatalities and the data from the 75+ age group only revealed 4. Therefore no statistically significant deviation appears (Table 2).

As far as rural versus urban area is concerned, urban area noted 57 (14%) excess deaths and the rural area experienced 80 (5%) excess deaths during the heat wave periods, but this was statistically insignificant for both areas (Table 2).

### 3.2. Analysis of Heat Waves in 2003

Overall in 2003, there were 88 (6%) more deaths than expected in Slovenia during the heat wave periods, but this was not statistically significant. None of these excess deaths occurred due to circulatory diseases and 25 (24%) of deaths occurred due to respiratory diseases. Examining deaths occurring due to other diagnoses, reveal excess deaths in all of the observed diagnosis subgroups, but not all were significant: endocrine diseases: 9 (21%), digestive system diseases: 14 (4%), genitourinary system diseases: 5 (36%), neoplasms: 35 (8%) (Table 2). In examining overall deaths by sex in 2003, there were 36 (4%) more male deaths than expected during heat waves and 52 (7%) more female deaths which proves to be insignificant for both sexes. No more circulatory disease related deaths than expected during the heat waves were discovered, neither for males, nor for females. In examining deaths occurring in males due to respiratory diseases, it was discovered that there were two (3%) more deaths than expected during the heat waves, and 23 (51%) more deaths among females than expected during the heat waves. Again this data proves to be analytically insignificant. Confidence intervals are too wide to draw any conclusions about differences between subgroups; excess mortality of males and females (Table 2).

An examination of overall deaths by age groups in 2003 revealed there were 44 (5%) more deaths than expected during heat waves in the age group of 5–74 years and 44 (6%) more deaths than expected during heat wave in the age group 75+ years. This was of no significance for either of the age groups. While examining deaths occurring in the two age groups due to circulatory diseases no more deaths than expected during the heat waves were discovered; neither for the 5–74 year group, nor for the 75+ years of age group, again playing an insignificant role. Examination of deaths due to respiratory diseases, occurring in the 5–74 years age group three more deaths or (18%) than expected during the heat waves were discovered. However 19 (27%) more deaths were noted among the members of the 75+ year age group, which is again, not a significant deviation. In examining deaths occurring due to circulatory diseases in males, there were no more deaths than expected during the heat waves discovered in neither the 5–74 group nor the 75+ age group. Analysis of deaths occurring in the 5–74 age group due to circulatory diseases in females showed there were 35 (69%) more deaths than expected during the heat waves and no excess deaths than expected during the heat waves occurred in the 75+ age group. This is an important mortality factor for females with circulatory system diseases in the 5–74 age group (RR = 1.69, 95% CI 1.08–2.62). Confidence intervals are too wide to conclude anything about the differences between subgroups: excess mortality of the two age groups (Table 2). Urban areas noted 13 (4%) excess deaths while rural areas noted 75 (6%) excess deaths during the heat wave periods. However this is statistically insignificant for both (Table 2).

### 3.3. Analysis of 2015 vs. 2003

When comparing all the subgroups in-between 2015 and 2003, we actually obtained some interesting results. Statistically significant higher numbers of deaths occurred in 2015 in comparison to 2003 in five subgroups: all, circulatory system diseases (RRR = 1.25, 95% CI 1.01–1.55); males, circulatory system diseases (RRR = 1.85, 95% CI 1.41–2.23); all, age group 75+ circulatory system diseases (RRR = 1.34, 95% CI 1.07–1.69); males, age group 75+, circulatory system diseases (RRR = 1.52, 95% CI 1.03–2.25); female, age group 75+, circulatory system diseases (RRR = 1.43, 95% CI 1.08–1.89) (Table 2).

### 3.4. “Lag” and “Harvesting” Effects

In our study, we have done several tests on individual heat waves actually, but neither “harvesting effect”, nor “lag effect” occurred after the tested almost every heat wave period. As a consequence we did not account the number of deaths in the lag days.

## 4. Discussion

Extreme heat waves affected Slovenia during the summers of 2003 and 2015, accompanied by a significant rise in short-term excess mortality. When considering our conclusions regarding all analysed heat waves of 2015 and 2003 in our country, it is important to bear in mind that this study is based on a very small number of the observed daily deaths and also these results strongly depend on the definition of heat waves, which internationally is not strictly agreed upon.

Especially in 2015, the heat waves impact appeared to be amplified among those aged over 75 years and among those with previous and acute circulatory diseases. A special phenomenon in 2015 occurred in the subgroup with respiratory system diseases where no excess deaths were noticed. On the other hand, in 2003 no excess deaths were noticed in the subgroup with circulatory diseases, but in the subgroup with respiratory system diseases many excess deaths occurred. As we defined 0 day “lag” and/or “harvesting” on the basis of tests, this implicates that not only a frail individuals were affected by the exposure, but also that the heat waves are real public health problem in Slovenia [43].

Old age is a very well-known risk factor for increased mortality during heat waves and it has been confirmed in many studies, like in our study in 2015 [14,15,45]. In the year 2008 the age group 60+ years represented 25% of Slovenia’s population. By 2050 the 35% of population is expected to be older than 60 years in Slovenia [46]. Elderly vulnerability is attributable to physiological and social factors.

Social factors include the following: living alone, income loss, multiple comorbidities, limited access to medical care, and lack of cooling [15,45]. In Slovenia in 2015 the proportion of social exclusion was 12.6% of men 65+ years of age compared with 25.5% of women of that age [47]. In 2003 15.8% Slovenian men 65+ years of age lived in poverty compared with 23.8% of women of that age [48], and in 2015 10.2% Slovenian men 65+ years of age lived in poverty compared with 22.8% of women of that age [49]. What is of concern is that social exclusion decreased more for man than for women and poverty is not distributed equally across Slovenia. There are statistical regions which are affected more and deserve special attention in public health interventions: Posavje region and Zasavje region [50]. As far as access to medical care is concerned, primary health care services in Slovenia are organized locally, such that they are equally accessible to all people without discrimination. Everyone must be assured continuously accessible urgent medical attention and emergency services. Compulsory health insurance is mandatory for all citizens with permanent residence in Slovenia, whereby everyone is bound to pay contributions under the solidarity principle [51].

Some physiological limitations appear during the normal aging process. However during heat waves, when extreme outdoor temperatures are present some impairment can be accelerated (e.g., blood distribution, sweating response during exposure to extreme temperatures) [15,45].

As far as circulatory diseases are concerned, in the year 2003 more than 50% adult Slovenians reported to have hypertension [52] and the number increased in the year 2015 and reached 56% of total population [53]. The trend for the future shows it will still increase as there are many persons with unrecognised hypertension [53]. The risk factors for hypertension are: being overweight, intake of alcohol and salt, sedentary lifestyle, high cholesterol levels, and others [52]. Hypertension is accompanied by elevation of peripheral resistance, by hypertrophy of the vascular smooth muscle [54] and vascular rarefaction [55]. This impairments lead to weakened core temperature regulation, as a consequence of impairments in the control of blood flow in the skin [56]. High blood pressure and elevated levels of cholesterol and triglycerides in the blood are well known risk factors for atherosclerosis [57]. The consequences of hypertension accompanied with atherosclerosis can be heart failure, acute coronary syndrome, cerebral stroke, chronic kidney diseases [52]. These effects may be exacerbated by medications, such as some psychotropic drugs and cardiac medications, which affect thermoregulatory capacity [58]. Physiological cardiovascular impairment in older individuals can make them more sensitive to elevated temperatures during heat waves. During heat waves blood flow must be redistributed toward periphery (vasodilatation), away from the core organs and an increase in sweat production [59]. Older individuals with pre-existing heart diseases have impaired mechanisms to increase their cardiac output sufficiently, and consequently the skin blood flow is not adequate during elevated core temperatures [60,61]. As body core temperature increases, dehydration occur which additionally affect heart and other organs [62]. It is estimated that impairments on core temperature regulation leads to increased blood viscosity due to dehydration [59,63]. Combined effect of pulmonary inflammation (described later) and hemoconcentration lead to acute coronary syndrome and cerebral stroke [63,64]. In Slovenia about 40,000 adult individuals have diagnoses of heart failure and the number increases with years. In 2015 more than 10% Slovenian population 70+ years of age experienced heart failure [65]. We do not have data on other heart diseases in Slovenia like: coronary and vascular heart diseases, cardiomyopathies, congenital heart defects and cerebrovascular and peripheral vascular diseases.

The heat effect was more noticeable in females in the subgroup of circulatory diseases, significantly in age group 5–74 in 2003. On the other hand, in the age group 75+, which mostly consists of females, as they generally outlive males, and in all age groups represented in 2015, we could not find such associations in Slovenia. Nevertheless in some prior studies, this phenomenon was evident [66]. It is hard to explain why females die more frequently in Slovenia during heat waves, as there is not physiological explanation yet, even though some reports propose a cultural, religious, and socio-economic explanation [66]. One of the reasons can be, that a higher proportion of older women, compared with older men, live below the poverty level, but obviously this is not a cause in Slovenia for 2003. In 2003 10.2% Slovenian men 16–64 years of age lived in poverty compared with 10% of women of that age [48]. According to the WHO report on heat waves vulnerabilities, older women are more affected than men in Europe [67].

When considering urban and rural number of deaths, we have to mention one protective factor. We live in a very green environment; even in urban areas. As an example it is worth mentioning that the capital of Slovenia, Ljubljana, has won the European Green Capital Award for 2016 [68]. Even though we did not confirmed statistically significant change for all population in urban area, it still remains a question what about age groups? Do older individuals in urban areas die more during heat waves in Slovenia? We did not answer on this question as there are too few daily deaths in Ljubljana and Maribor to conclude anything about differences in these subgroups. We will try to explain this by reviewing mortality in a decade to get a higher number of deaths.

Other results of our study differ in some respect from other reported studies of mortality on days of high heat. In opposition to many of the previous studies [9,17,18,19,20], we did not find statistically significant proof of increase in deaths during heat waves due to respiratory diseases, endocrine disease, digestive system diseases, genitourinary system diseases and neoplasms.

The lungs of older individuals undergo physiological changes with age that can impair breathing, even without disease [69]. Older individuals are often affected by compromised immune systems [69]. Lung changes during heat waves are mainly due to changes in air quality. Heat exposure can trigger inflammation mainly due to elevated ground-level ozone exposure [70]. In Slovenia this is especially relevant for the small town of Nova Gorica on the west and for the Slovene littoral. Older individuals are more sensitive to infections and pathogens [69,71]. Facilitated spread or emergence of vector-, water-, and food-borne diseases is characterized for heat wave periods [72]. Pulmonary infection or inflammation can accelerate growth of atherosclerotic plaques [64]. There are also evidences that inflammation in respiratory tract promote hyper-coagulation through different mechanisms [73]. These mechanisms, along with already impaired cardiovascular response, promote thrombotic events [63,64]. Inflammation itself, also worsen chronic obstructive pulmonary disease and asthma, which is highly prevalent in elderly [74]. It is estimated, that about 10% of Slovenians suffer from chronic obstructive pulmonary disease in 2013 [75] and 16% from asthma in 2015 [76], and the prevalence increases, especially in undeveloped areas of Slovenia.

The prevalence of diabetes type 2 is increasing in Slovenia and poorer region like Zasavje, Posavje and Podravje are the most affected. In 2015 there were 106,318 (6.8%) patients with type 2 diabetes in Slovenia and this number is probably underestimated, since many diabetics remain undiscovered and untreated. Since the last decade, 3% of new cases appear every year. It is more prevalent among men than among women and in the age group 65+ years [77]. Diabetes and poor glucose control impair ability of blood vessels in the skin to dilate and decrease the amount of blood flow in the skin surface, reducing dissipation of heat [78,79]. The presence of neuropathy, which is highly relevant for type 1 diabetics, affects sweating response, especially in distant region [80], but the exact mechanism is still unknown. Elevated temperatures may also affect metabolic alterations which reduce heat tolerance [81].

Obesity is a very well known risk factor for type 2 diabetes and is increasing relatively fast all over Europe. Obese individuals itself have lower sensitivity to heat stress, but exact mechanisms for this still poses many questions [82]. On the other hand, a smaller ratio of body surface area to body mass reduces ability to sweat [15].

Heat stress results in the redistribution of blood from the splanchnic and renal vasculature to the periphery what affect renal blood flow and cause renal impairments [83]. The situation is aggravated by already chronic renal diseases associated with ageing.

Despite the fact that in many studies neurological and mental diseases contributed highly to the burden of disease during heat waves, the effects on elderly mental health during heat waves are severely lacking. It is estimated that socio-economic status and demographic factors, like marriage, divorces, migrations, play an important role in the number of deaths due to mental and nervous system diseases [17,19,20]. In Slovenia, in general the incidence of suicide is slowly decreasing, but still remains highly and above the European average. It is estimated to be around 20/100 of population in one year and the ratio between man and women is 3.8:1. Poorer regions like Podravska, Posavska, Pomurska are also more affected in Slovenia. In Slovenia suicide is mainly a consequence of unrecognised or untreated mental illnesses like depression and schizophrenia [84]. In our study we have not done analysis for mental and nervous system diseases, as there are too few deaths due to these causes and on the basis of these small number we could not estimate how diverse factors (socio-economic status, demographical data) influence deaths during heat waves.

When considering our conclusion about the differences between subgroups in 2015 and 2003, it is important to bear in mind that in the period between 2003 and 2015 heat waves have become more frequent, more intense, and have come to last longer. As we noticed in 2015, heat waves lasted longer and they were accompanied by higher average of the average pseudo-equivalent temperatures. People live longer, so the global burden of chronic and degenerative disease increases. The life expectancy at birth slowly increases also in Slovenia as well (2003: 72.6 for males and 80.4 for females; 2015: 77.59 for males and 83.51 for females) [85]. Consequently, these two reasons potentially contributed to more deaths due to circulatory diseases in 2015.

As far as adaptation is concerned, people over 70 years of age, suffering from cardiovascular diseases, pulmonary diseases, long standing diabetes types 1 and 2, and obesity are at increased risk of heat-related stress during heat waves, as physiological impairments occur in the different thermoregulation mechanisms described. If we know these mechanisms, we still can empower elderly individuals to cope properly with prevention on days of high heat (appropriate behaviour, especially fluid intake, careful medication use). Social factors are the following: sedentary lifestyle, living alone and social exclusion, decreased mobility, which can also contribute to an increased risk of heat-illness [15]. We can influence on this factors with different protective approaches: to identify lonely old age individuals, provide air conditioners and fans for them, moving them to cool environments during prolonged heat events and control their medical situation.

The practically applicable results of this study are: the heat wave-associated deaths increased in 2015 compared to 2003; the most vulnerable population during heat waves in Slovenia are the elderly (75 years of age and more); Elevated RRR when comparing 2015 and 2003 for specific causes of death in heat waves contributes to identification of vulnerable subgroup (persons with circulatory system diseases), for further confirmation it would be interesting to consider morbidity.

We are aware that our results are limited because of small sample group, and evidently from Table 2 confidence intervals are very wide in this analysis, so any interpretation is burdened by speculation and incomplete answers. This is partially due to the small population of Slovenia. On the other hand, only underlying causes of deaths were recorded and it would be interesting to know if any other diseases, injuries, conditions, or events contributed to the death toll. One other limitation of the study is that we did not consider confounding factors, which are also associated with elevated deaths during summer. Specifically: summer oxidation smog, other outdoor air pollutants and indoor living conditions and socio-economic status.

## 5. Conclusions

Even though Slovenia is a small country in the middle of Europa, it is important to know how heat waves affect the population’s health. The results of these analyses show that we must be especially careful in the future. The most vulnerable groups, which are old people and those with circulatory diseases, increased in 2015 compared to 2003. As people will live longer and obviously in Slovenia all chronic non-communicable diseases increase, especially in poorer regions, and the heat waves will be more pronounced, the burden of deaths during heat waves will consequently increase over the next years. To stop this trend additional public health interventions are needed. First of all, we can influence social factors for adaptation described, and also warn members of vulnerable population to increase physiological factors of adaptation. Heat waves are included on the list of dangerous weather events and national weather service issue warnings in case of occurrence of heat waves. Reaching vulnerable population with mass-media is sufficient in Slovenia due to the size of its citizens. We use internet publications to inform the entire population about preventive measures meant for elder individuals and those with circulatory diseases when facing heat waves. We also use country-wide and local TV channels, radio stations and newspapers to publish some useful information about how to cope with elevated temperatures. Primary care centres are aware of the problem and warn chronically ill patients, e.g., patients on antihypertensive drugs of possible complications. In the future, we intend to have some workshops around the country to reduce the impact of heat waves on population health.

## Figures and Tables

**Table 1:** Heat waves periods and pseudo equivalent temperature in Slovenia between 1 May and 30 September in 2015 and 2003.

Heat Waves in Slovenia in 2015	Average Pseudo Equivalent Temperature (°C), 2015	Heat Waves in Slovenia in 2003	Average Pseudo Equivalent Temperature (°C), 2003
**June**		**June**	
3 June 2015	56.6	9 June 2003	58.2
4 June 2015	56.5	10 June 2003	60.5
**July**		11 June 2003	60.0
2 July 2015	59.4	12 June 2003	63.5
3 July 2015	61.4	13 June 2003	62.8
4 July 2015	60.3	14 June 2003	58.4
5 July 2015	62.5	15 June 3003	56.5
6 July 2015	68.4	23 June 2003	61.9
7 July 2015	67.7	24 June 2003	61.6
8 July 2015	69.5	25 June 2003	62
14 July 2015	57.7	30 June 2003	57.4
15 July 2015	61.1	**July**	
16 July 2015	66.2	1 July 2003	58.3
17 July 2015	69.1	16 July 2003	56.2
18 July 2015	67.9	17 July 2003	61.9
19 July 2015	67.9	21 July 2003	61.3
20 July 2015	64.7	22 July 2003	61.3
21 July 2015	67.8	23 July 2003	61.9
22 July 2015	66.5	**August**	
23 July 2015	69.7	4 August 2003	62.8
24 July 2015	65.6	5 August 2003	62.7
25 July 2015	64.3	6 August 2003	61.9
**August**		9 August 2003	59.3
2 August 2015	58.2	10 August 2003	59.7
3 August 2015	60.9	11 August 2003	57.2
4 August 2015	62.9	13 August 2003	62.7
5 August 2015	63.6	14 August 2003	62.7
6 August 2015	65.8	17 August 2003	60.9
7 August 2015	65.8	18 August 2003	64.6
8 August 2015	61.5	23 August 2003	58.1
14 August 2015	64.0	24 August 2003	56.9
15 August 2015	69.4	28 August 2003	56.0
16 August 2015	57.1	29 August 2003	61.5
27 August 2015	58.3		
28 August 2015	62.1		
29 August 2015	64.1		
30 August 2015	64.6		
31 August 2015	59.7		
**September**			
16 September 2015	61.6		
17 September 2015	61.3		
18 September 2015	59.7		
**No. of heat waves**	7	**No. of heat waves**	11
**No. of heat wave days**	39	**No. of heat wave days**	31
**Average of the average pseudo equivalent temperature during heat waves (°C)**	63.36	**Average of the average pseudo equivalent temperature during heat waves (°C)**	60.34

**Table 2:** Relative risks (RR) for death during heat waves, 95% confidence intervals (95% CI) and excess deaths by the observed diagnosis, sex, age groups, and area for 2015 and 2013, and relative risks ratio (RRR), 95% confidence intervals (95% CI) and *p*-value for comparing association between 2015 and 2003 for Slovenia.

Underlaying Cause of Death (ICD 10 Code)	2003				2015				2015 vs. 2003	
	Heat Wave Deaths	Reference Day Deaths/2	RR (95%CI)	Excess Deaths (%)	Heat Wave Deaths	Reference Day Deaths/2	RR (95%CI)	Excess Deaths (%)	RRR (95% CI)	*p*
ALL, all causes (A00–T98)	1603	1515	1.06 (0.97–1.15)	88 (6%)	1996	1859	1.07 (0.99–1.16)	137 (7%)	1.01 (0.90–1.13)	0.881
MALE, all causes (A00–T98)	845	809	1.04 (0.93–1.18)	36 (4%)	955	907	1.06 (0.95–1.19)	52 (6%)	1.02 (0.86–1.20)	0.826
FEMALE, all causes (A00–T98)	758	706	1.07 (0.95–1.22)	52 (7%)	1041	952	1.1 (0.98–1.22)	89 (10%)	1.03 (0.88–1.21)	0.749
age group 5–74 years (A00–T98)	804	760	1.05 (0.94–1.19)	44 (5%)	751	729	1.04 (0.91–1.17)	22 (4%)	0.99 (0.83–1.17)	0.267
age group 75+ years (A00–T98)	799	755	1.06 (0.94–1.19)	44 (6%)	1245	1130	1.11 (1.00–1.22)	115 (10%)	1.05 (0.89–1.22)	0.562
ALL, circulatory system diseases (I00–I99)	534	585	0.91 (0.75–1.05)	−49	783	685	1.14 (1.01–1.3)	98 (14%)	1.25 (1.01–1.55)	0.036
MALE, circulatory system diseases (I00–I99)	233	273	0.62 (0.52–0.75)	−40	319	277	1.15 (0.94–1.41)	42 (15%)	1.85 (1.41–2.43)	<0.001
FEMALE, circulatory system diseases (I00–I99)	301	312	0.96 (0.79–1.17)	−11	464	408	1.13 (0.96–1.34)	56 (13%)	1.18 (0.91–1.52)	0.215
ALL, age group 5–74 years (I00–I99)	186	187	0.99 (0.77–1.27)	−1	142	136	1.04 (0.78–1.39)	6 (4%)	1.05 (0.71–1.54)	0.803
ALL; age group 75+ years (I00–I99)	348	398	0.87 (0.73–1.04)	−50	641	549	1.17 (1.01–1.34)	92 (17%)	1.34 (1.07–1.69)	0.011
MALE, age group 5–74 years (I00–99)	133	137	0.97 (0.73–1.30)	−4	98	86	1.14 (0.79–1.63)	12 (14%)	1.17 (0.74–1.87)	0.497
MALE, age group 75+ years (I00–I99)	100	136	0.73 (0.54–1)	−36	221	191	1.11 (0.87–1.41)	24 (11%)	1.52 (1.03–2.25)	0.003
FEMALE age group 5–74 years (I00–I99)	86	51	1.69 (1.08–2.62)	35 (69%)	44	50	0.88 (0.53–1.44)	−6	0.52 (0.26–1.02)	0.056
FEMALE, age group 75+ years (I00–I99)	215	261	0.82 (0.66–1.02)	−46	420	358	1.17 (0.98–1.40)	62 (17%)	1.43 (1.08–1.89)	0.013
ALL, respiratory system diseases (J00–J99)	129	104	1.24 (0.90–1.71)	25 (24%)	105	105	1 (0.72–1.39)	0	0.81 (0.51–1.28)	0.363
MALE, respiratora system diseases (J00–J99)	61	59	1.03 (0.66–1.6)	2 (3%)	47	45	1.04 (0.63–1.73)	2 (4%)	1.01 (0.51–1.98)	0.984
FEMALE, respiratory system diseases (J00–J99)	68	45	1.51 (0.94–2.43)	23 (51%)	57	59	0.96 (0.62–1.53)	−1	0.64 (0.33–1.23)	0.177
ALL, age group 5–74 years (J00–J99)	38	35	1.08 (0.67–1.91)	3 (8%)	11	15	0.71 (0.29–1.86)	−4	0.66 (0.23–1.91)	0.441
ALL, age group 75+ years (J00–J99)	88	69	1.27 (0.86–1.89)	19 (27%)	94	90	1.04 (0.73–1.49)	4 (4%)	0.82 (0.48–1.39)	0.465
ALL, endocrine diseases (E00–E90)	51	42	1.21 (0.73–2.02)	9 (21%)	40	33	1.19 (0.66–2.11)	7 (19%)	1.75 (0.72–3.40)	0.254
ALL, digestive system diseases (K00–K96)	115	101	1.14 (0.82–1.58)	14 (4%)	77	83	0.96 (0.64–1.35)	−6	0.84 (0.52–1.37)	0.497
ALL, genitourinary system diseases (N00–N99)	19	14	1.36 (0.57–3.23)	5 (36%)	34	25	1.36 (0.71–2.64)	9 (36%)	1 (0.34–2.97)	0
ALL, neoplasms (C00–D48)	455	420	1.08 (0.92–1.28)	35 (8%)	651	618	1.05 (0.92–1.25)	33 (5%)	0.97 (0.78–1.22)	0.810
ALL, urban area (A00–T98)	355	342	1.04 (0.86–1.25)	13 (4%)	466	409	1.14 (0.96–1.34)	57 (14%)	1.1 (0.85–1.4)	0.478
ALL, rural area (A00–T98)	1248	1173	1.06 (0.96–1.17)	75 (6%)	1530	1450	1.05 (0.96–1.15)	80 (5%)	0.99 (0.87–1.13)	0.168
